# Complete resection of a giant mediastinal teratoma occupying the entire right hemithorax in a 14-year-old boy

**DOI:** 10.1186/1471-2482-14-56

**Published:** 2014-08-24

**Authors:** Honglin Zhao, Daxing Zhu, Qinghua Zhou

**Affiliations:** 1 Department of Lung Cancer Surgery, General Hospital, Tianjin Medical University, No. 154, Anshan Road, Heping District, Tianjin 300052, China

## Abstract

**Background:**

Mature teratomas are the most common histological type of germ cell tumors.

**Case Presentation:**

A 14-year-old boy was referred to our hospital with a giant mature teratoma occupying the entire right hemithorax compressed the superior vena cava (SVC) and total atelectasis of the right lung. He was misdiagnosed as malignant teratoma by a fine-needle biopsy in a hospital. After 4-cycle of chemotherapy without effect, he underwent an unsuccessful exploratory thoracotomy. Venous conduit bypass between the right jugular vein and right femoral vein was established in the operating room for superior vena cava (SVC) replacement if needed. En bloc resection of the huge tumor, wedge resection of the dense adhesions of the right lung and partial pericardectomy were successfully performed, and lung function was recovered.

**Conclusion:**

To the best of our knowledge, this is the first report of complete resection of the teratoma occupying the whole right hemithorax combined with wedge resection of the right upper, middle and lower lobes and partial resection of the pericardium.

## Background

Mature teratomas are the most common histological type of germ cell tumors. Germ cell tumors are predominantly found in gonads, while the anterior mediastinum is the most common extragonadal site. In the article, we have found a giant mediastinal teratoma occupying the entire right hemithorax in a 14-year-old boy by complete resection.

## Case report

**Figure 1:**
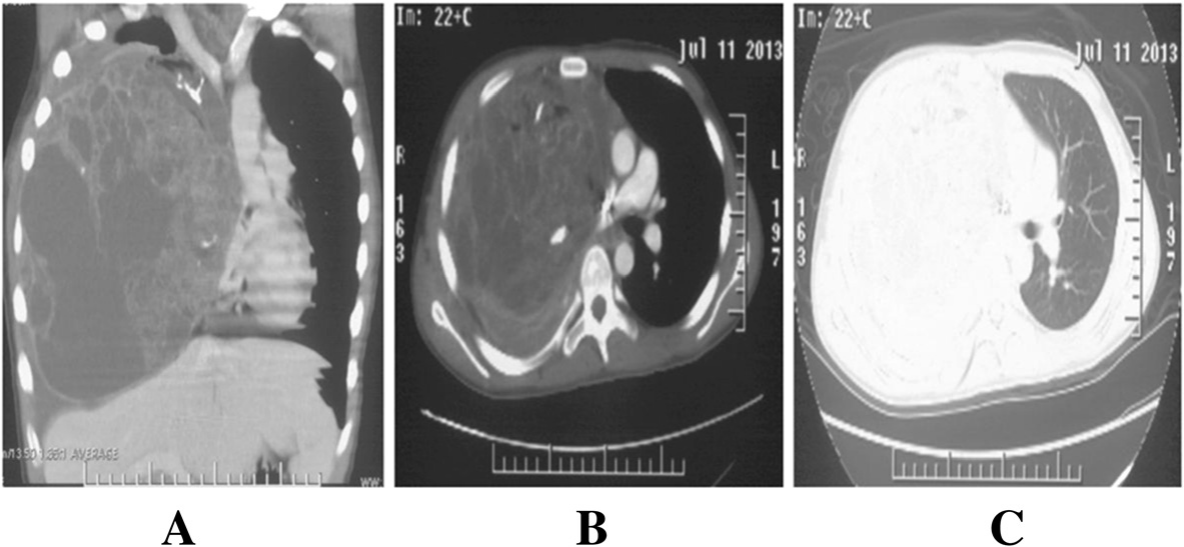
**Enhanced chest CT scan before operation. (A)** In the coronal mediastinal window, preoperational CT scan showing the mass compressing the mediastinal vessels, heart and airway. **(B)** In the mediastinal window. **(C)** In the lung window.

**Figure 2:**
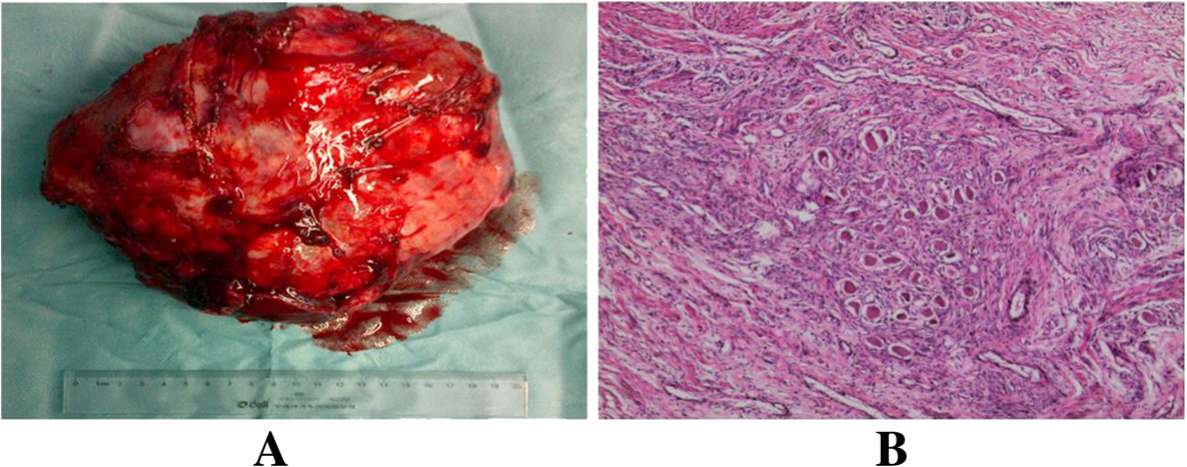
**The images of surgical specimen and histopathology. (A)** Surgical specimen’ largest diameter was 20 cm (smaller than preoperational CT scan after fluid aspiration) and it weighed 4320 g. **(B)** Hematoxylin and eosin (H&E) staining of terotoma.

**Figure 3:**
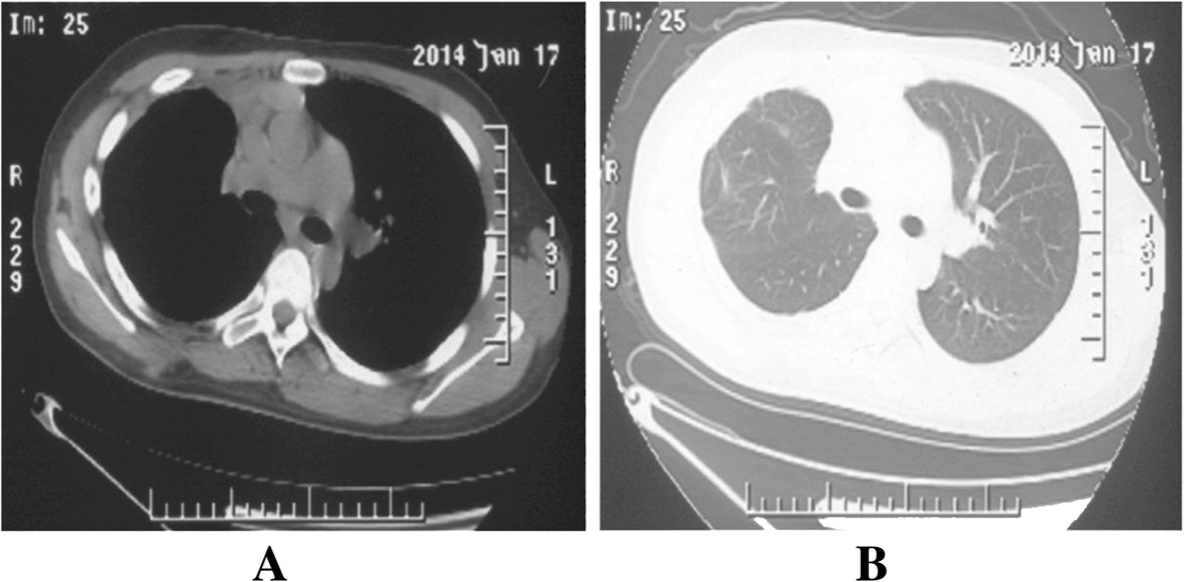
**CT scan after operation. (A and B)** CT scan 6 months after operation.

A 14-year-old boy complained of short of breath for 10 months was admitted to our hospital. Computed tomography (CT) revealed a giant multilocular mass of approximate 20 × 20 × 25 cm in size with right pleural effusion. It compressed the heart, superior vena cava, and diaphragm with the total right lung atelectasis. The tumor contained multiple cysts with calcifications and fat. Tumor cells were not found in the pleural effusion (Figure 1). The teratoma was considered unresectable and malignant, which contained immature components by CT guided fine-needle biopsy 8 month ago in a foreign hospital. The boy took 4-cycle BEP chemotherapy. But the tumor didn’t shrink evaluated by CT scan. Then the patient received an exploratory thoracotomy 3 month ago by another hospital. But the huge size, widespread adhesions and abundant new vascularization of the tumor resulted in massive bleeding allowed only a small part of the mass excised for pathologic diagnosis. Pathologic findings revealed a mature teratoma. Cranial MRI, upper abdomen CT scan and bone nuclear scan revealed no further lesions in our hospital. The right-sided posterolateral thoracotomy was performed on July 18, 2013. Venous conduit bypass between the right jugular vein and right femoral vein was established immediately before operation in case of SVC replacement. Because of the widespread and severe adhesions among the tumor with surrounding chest wall, diagram, lung, pulmonary vessels, SVC, and pericardium, great care was taken for the dissection. Reducing the tumor volume to facilitate the dissection, the fluid contents of the mass were aspirated via a small incision of the wall. With partial resection of the dense adhesions of the pericardium, and wedge resections of the severe adhesions of the right upper, middle, and lower lobes of the lung, en bloc resection of the huge tumor was completed (Figure 2). The tumor weighed 4320 g and its largest diameter was 25 cm. Considering the weight of the tumor, continued traction via the sutures placed on the tumor maintained during dissection to prevent from compressing of the heart. The right lung was re-expanded. We used ventilation with peep 6 cm on the first postoperative day, 3-day corticosteroids, and diuretics to prevent re-expansion pulmonary edema. The postoperative course was uneventful, and the boy was discharged from the hospital on the 16th postoperative day (Figure 3). Final pathological diagnosis was a benign mature teratoma. At 6-month follow-up, the patient is well without recurrence.

## Discussion

Mature teratomas are the most common histological type of germ cell tumors. Germ cell tumors are predominantly found in gonads, while the anterior mediastinum is the most common extragonadal site [1,2]. Because of the early asymptomatic, teratomas were always got longer course, larger tumors and compression of surrounding organs. Some teratomas contain malignant components, but the effect of chemotherapy is poor. Surgical resection is the only effective way to treat teratoma, especially for mature teratoma.

The choice of incision for removing the giant mediastinal tumor depend on the tumor size, location, the relationships between the tumor and the associated vital structures, and the surgeon’s experience. In our case, right posterolateral access was chosen, as the tumor was almost entirely located into the right hemithorax, reaching and adherent to the left hemi-diaphragm. And contrast CT scan showed no signs of invasion of left brachial vein. As several literature reported, we agree that reducing the tumor volume by fluid aspiration via a small incision of the tumor wall facilitate the dissection for removing the huge mediastinal tumors [3-6]. During dissection of the giant mediastinal tumors, continued traction the tumor via the sutures placed on the tumor can prevent from compressing of the heart to avoid circulatory collapse. There are usually widespread and severe adhesions between huge mediastinal tumors and the adjacent structures. Great care must be taken during dissection as the damage of vital structures can induce fatal cardiopulmonary complications. As reported in our previous report, partially resection of the infiltrate pericardium or diaphragm is safe and helpful for dissection [7]. We routinely set up the venous conduit bypass between the right/left jugular vein and right/left femoral vein before SVC replacement to preventing from high venous pressure when SVC clamped [8]. As contrast CT scan showed the tumor severely adhered to SVC, we established the venous conduit bypass between the right jugular vein and right femoral vein before operation in case of SVC replacement. Although the division between the tumor and SVC was successful in this case, the venous bypass provided a safe support for surgery.

## Conclusion

In conclusion, we report an unusual case of the teratoma occupying the whole right hemithorax in a 14-year-old boy, and the tumor was completely removed and combined with wedge resection of the right upper, middle and lower lobe and partial resection of the pericardium.

## Consent

Written informed consent was obtained from the patient for publication of this case report including associated images and video.

## Competing interests

The authors declare that they have no competing interests.

## Authors’ contributions

All authors were involved in the preparation of this manuscript. HZ and QZ performed the operation, collected the data and wrote the manuscript. DZ performed the operation and designed the study. HZ summarized the data and revised the manuscript. All authors read and approved the final manuscript.

## Pre-publication history

The pre-publication history for this paper can be accessed here:

http://www.biomedcentral.com/1471-2482/14/56/prepub
